# Supplementary material for: HPV DNA screening and vaccination strategies in Tunisia
Source: Sci Rep. 2025 Jul 31;15:27916. doi: 10.1038/s41598-025-13423-3 (PMC12313955; doi:10.1038/s41598-025-13423-3)
Supplement: Supplementary file 1 — Supplementary Information. [file 41598_2025_13423_MOESM1_ESM.pdf]

# Appendix-Supplementary Material to HPV DNA Screening and Vaccination Strategies in Tunisia

Anas Lahdhiri, Beya Benzina, Emna Ennaifer, Haifa Tounsi, Ahlem Gzara, Soumaya Rammeh-Rommani, Oumaima Laraj, Heger Arfaoui, Robyn Stuart, Amira Kebir and Slimane BenMiled

July 10, 2025

## 1 Genotype Identification

In the context of our simulation, it is essential to accurately identify and model the different genotypes of the HPV virus and their distribution within the Tunisian population. We based our study on a survey conducted in Tunisia in 2013, aimed at determining the distribution of HPV genotypes in the country. The genotypes identified in this survey included the following: HPV-6, 11, 16, 18, 31, 35, 39, 40, 42, 43, 44, 52, 53, 54, 56, 58, 59, 62, 66, 68, 70, 75, 81, 84, 89. It is important to note that these genotypes include both high-risk types, i.e., those that can cause cancer, and low-risk types, which do not cause cancer. To better understand their potential impact on public health, it is crucial to know the used classification to classify substances according to their carcinogenic potential. Here is an explanation of the different classes :

- Group 1 Carcinogens: This category includes substances that are recognized as carcinogenic to humans. The HPV genotypes included in this category are those most closely associated with the development of cervical cancer
- Group 2A Carcinogens: Substances in this category are probably carcinogenic to humans. There is sufficient evidence of their carcinogenicity in animals and limited evidence in humans
- Group 2B Carcinogens: Substances in this category are possibly carcinogenic to humans. There is limited evidence of their carcinogenicity in humans and insufficient evidence in animals
- Phylogenetic Analogies with Carcinogenic Types: This classification includes HPV types that, although not directly proven to be carcinogenic, have phylogenetic similarities with known carcinogenic types
- Non-Carcinogenic: This category includes HPV types that have not been associated with a cancer risk

Applying this classification, we first eliminated the genotypes known to be non-carcinogenic. Then, using the classification presented in appendix table

1, derived from an in-depth study of HPV genotypes responsible for cancer, we refined our selection. Ultimately, we retained the following carcinogenic genotypes for our simulation: HPV-16, 18, 31, 35, 39, 52, 56, 58, 59. The other genotypes were not completely excluded from the model but were grouped into a cluster representing low-risk genotypes

Appendix Table 1: Classification of HPV genotypes using the classification found in [1]

| **Carcinogenic Potential Class** | **Genotypes** |
|---|---|
| Group 1 | 16, 18, 31, 33, 35, 39, 45, 51, 52, 56, 58, 59 |
| Group 2A | 68 |
| Group 2B | 26, 53, 66, 67, 70, 73, 82 |
| Phylogenetic Analogies | 30, 34, 69, 85, 97 |

The main challenge we faced was that the HPVsim model initially only allowed the selection of HPV-16 and HPV-18 genotypes. Therefore, we had to modify the source code of the HPVsim model to include the newly identified genotypes while ensuring no errors or bugs were introduced. With these modifications, our model can now more accurately represent the diversity of HPV genotypes present in Tunisia. This improves the precision of our simulations and provides more reliable results regarding the transmission and evolution of the virus, which is crucial for evaluating the potential impact of the vaccination and screening strategies we aim to implement.

# 2 Model Parameterization

## 2.1 Choice of Simulation Parameters

Multi-agent models and graph-based models are used to represent complex systems where the interactions between agents or nodes are dynamic and often influenced by random processes. Due to this random component, these simulations are inherently stochastic, meaning that the results can vary from one execution to another. To mitigate uncertainty, it is common to do large number of replicates or/and increase the number of agents. This allows for capturing the inherent variability and providing a more accurate estimation of the average results. For this we conducted an analysis where we tested different agent populations 10000, 50000, 100000 and different numbers of replicates: 1, 2, 3, 5, 10, 15, 20. We run HPVsim with the desired number of replicates 10 times, and at each run we calculate the mean and standard deviation of cancer cases resulting from running that number of replicates. We define the centroid as the average value we get from the 10 runs. This process was repeated for every agent population size we set to test.

The analysis on the mean of cancer cases outcomes showed that for a population of 10000 agents the position of the centroid of the scatter plot becomes stable starting from 5 replicates, although the dispersion of the scatter plot becomes significantly reduced at 15 replicates (see Appendix Figure 1a). For a population of 50000 agents, the centroid stabilizes at 5 replicates, and the dispersion of the scatter plot becomes significantly less dispersed also at 5 replicates (see Appendix Figure 1b). Regarding a population of 100000 agents, the centroid is stable even at one replicate, but dispersion of the scatter plots reduces only after 5 replicates (see Appendix Figure 1c). To summarize, to ensure mean stability and minimize disparity (i.e., standard deviation of means), it is recommended to perform 15 replicates for a population of 10000 agents, 5 replicates for population of 50000 agents and 5 replicates for population of 100000 agents.

The analysis on the standard deviation of cancer cases outcomes showed that For a population of 10000 agents, centroid stabilizes at 10 replicates, but the scatter plot remains dispersed even with 20 replicates (see Appendix Figure 2a). For a population of 50000 agents, the centroid stabilizes after 10 replicates, and the dispersion is also reduced at 10 replicates (see Appendix Figure 2b). For a population of 100000 agents, the standard deviation is stable after 10 replicates however the dispersion is reduced after 5 replicates only (see Appendix Figure 2c). To ensure standard deviation stability and minimize disparity (i.e., standard deviation of standard deviations), it is recommended to perform 20 replicates for a population of 10000 agents and 10 replicates for populations of 50000 or 100000 agents.

As a result of all the above, we have identified three options in order to ensure statistically stable results: 30 replicates for a population of 10000 agents, or 10 replicates for populations of 50000 or 100000 agents. Therefore to choose between the valid options it's crucial to consider runtime. Appendix Figure 3 shows the evolution of running time as the number of replicates increases for each agent population size. It is clear that 10 replicates for 50000 agents take much less time than 10 replicates for 100000 agents.Although 20 replicates for 10000 agents take almost as much time as 10 replicates for 50,000 agents, we

opted for 10 replicates with 50,000 agents, as this configuration ensures a better statistical stability.

### 2.1.1 Calculation of Age of Sexual Debut

The age of sexual debut was derived from data collected in the 2022 survey conducted by the Tawhida Ben Cheikh Group, focusing on unmarried Tunisians aged 18 to 30. Initially, we calculated the average age of sexual debut reported by survey participants, which included both men and women. To adjust for broader population dynamics, particularly considering marriage norms, we weighted these averages using marriage age data provided by the National Institute of Statistics for young Tunisians (aged 18 to 30). This adjustment aimed to more accurately reflect when individuals typically initiate sexual activity in relation to marriage. As a result, the adjusted average age of sexual debut was determined as 23 years for women and 22 years for men. These calculations form the foundation for modeling sexual behavior dynamics in our study, acknowledging the influence of cultural norms and survey limitations that may affect the precision of these estimates.

## 2.2 Calibration and Validation

The genotype distribution data we have is from the year 2013 [2]. Since our model begins the simulation in 1990, we cannot simply input this data directly as the initial distribution. Given the dynamic nature of the model, the genotype distribution evolves over time based on the interactions and parameters of the model. Therefore, it is necessary to define a different initial distribution that, after simulation, will correspond to the observed distribution in 2013. To achieve this, we manually adjusted the initial distribution of genotypes. This process involved numerous iterations and fine adjustments until the resulting distribution in 2013 satisfactorily matched the observed data. The results of this calibration are presented in Appendix Figure 4.

Calibration was performed on data of yearly CC death in Tunisia from 2020 [3] and 2022 [5]. To calibrate our model, we adjusted several parameters whose values were uncertain due to a lack of specific data. These parameters include: transmission probability (beta), relative transmissibility from males to females in penetrative sex (transm2f), relative transmissibility from females to males in penetrative sex (transf2m), male infidelity rate (m_cross_layer) and female infidelity rate (f_cross_layer). HPVsim includes a calibration tool based on random sampling of values and uses the mean absolute error as a fitting metric. However, this default algorithm has limitations, including non-guaranteed convergence and very long computation times. To overcome these limitations, we developed our own calibration function using the optimized stochastic gradient algorithm ADAM. This algorithm also uses mean absolute error as the performance metric. Thanks to this approach, we were able to achieve and efficient convergence. The results of this calibration are illustrated in Appendix Figure 5, which shows that the forecasts generated by HPVsim after calibration are within a 10% margin of error compared to national forecasts for new cancer cases, and the curve has almost the same slope as the curve of cases from the national predictions. This accuracy confirms the effectiveness of our calibration method and ensures that the model reliably reflects current and future cancer case trends in Tunisia. HPV prevalence and CC case for validation were used to validation.

# 3 Current HPV Screening and Treatment Process

## 3.1 Deriving the Annual Screening Probability from Overall Coverage

Statistics have shown that the HPV screening coverage rate among women aged between 35 and 60 years of age is 15%[8]. This coverage is not the result of a state strategy to combat cervical cancer but is the result of spontaneous screening carried out by the patient herself or on medical prescription by the doctor. It is necessary to transform this coverage percentage into a yearly probability of screening to be used in the HPVsim model. We have a screening window for women between the ages of 35 and 60. Women enter and exit this age group as they age, so the time spent in the screening window varies uniformly. The annual probability of getting screened is $p$. Thus the probability of being screened at least once is :

$$P(\text{Screen at least once}) = 1 - (1-p)^t$$

In this case the coverage rate is :

$$\text{Coverage Rate} = \frac{1}{25}\sum_{t=0}^{25}\left(1-(1-p)^t\right)$$

In this equation for an annual probability of screening $p = 1.33$ we obtain a coverage rate of $\approx 15\%$.

### 3.1.1 Screening and Treatment Process

HPV screening in Tunisia follows a well-defined clinical process. Screening begins with a Pap test, also known as a cervical smear test. This test involves collecting cells from the cervix and examining them under a microscope to detect abnormalities. The main goal of the Pap test is to early identify cellular changes that could develop into cervical cancer, thus allowing for quick and preventive intervention. When Pap smear test results reveal cellular abnormalities, the patient is referred for a colposcopy with a biopsy. A colposcopy is a procedure where a colposcope is used to closely examine the cervix for suspicious areas. A biopsy, which involves taking a small tissue sample, is performed for a more detailed histological analysis. The results of the colposcopy and biopsy help determine the type of lesions present:

- LSIL (Low-grade Squamous Intraepithelial Lesion): LSIL are mild cellular changes, often caused by a transient HPV infection. Although these lesions have a low potential to become cancerous, they require monitoring. The typical treatment for LSIL is ablation, a procedure that removes or destroys the abnormal tissue using a laser.
- HSIL (High-grade Squamous Intraepithelial Lesion): HSIL indicates more severe cellular changes that have a higher risk of progressing to cervical cancer. HSIL treatment involves a conization procedure, which removes a cone-shaped portion of the cervix containing the abnormal cells.

- Cancer: If the biopsy reveals the presence of cancer cells, the patient is immediately referred for appropriate cancer treatment, which may include a combination of surgery, radiotherapy, and chemotherapy, depending on the cancer's stage and location.

This systematic process helps identify and treat cellular abnormalities at an early stage, thus reducing the progression to more severe forms of the disease.

## 3.2 Vaccine Cross-protection

This appendix provides the updated cross-protection values (Appendix Table 2) of Bivalent vaccine against different HPV genotypes using one dose. We assumed a 100% efficacy for the targeted genotypes by a given vaccine, although this may be overestimated, we believe it won't affect the outcomes of the study as the vaccination intervention will be the same across the tested screening strategies. We also assumed a 0% efficacy in case we didn't find any evidence of protection.

Appendix Table 2: Cross-protection of different HPV vaccines against various genotypes

| **Vaccine** | **Genotypes** | | | | | | | | |
|---|---|---|---|---|---|---|---|---|---|
| | 16 | 18 | 31 | 35 | 39 | 52 | 56 | 58 | 59 | low-risk |
| Bivalent | 100% | 100% | 73% | 0% | 0% | 17% | 26% | 5% | 0% | 0% |

## 3.3 Interventions Costs

This appendix offers a detailed summary of unit costs (Appendix Table 3). It outlines the expenses associated with various healthcare interventions for HPV infections and cervical cancer, reflecting 2024 pricing and applying a 3% discount rate for future years.

Appendix Table 3: Summary of Healthcare Intervention Unitary Costs. CNAM referred to the Tunisian National Health Insurance Fund

| **Healthcare Intervention** | **Unitary Cost (USD) in 2024** | **Source** |
|---|---|---|
| Bivalent vaccine (per dose) | 10.25 | Source [10] |
| Cervical cancer treatment (per case) | 2463.00 | Source [6] |
| Pap smear test | 6.50 | CNAM Hospital Price Template |
| Colposcopy | 6.80 | CNAM Hospital Price Template |
| Pre-cancer treatments (excision) | 18.00 | CNAM Hospital Price Template |
| Pre-cancer treatments (ablation) | 18.00 | CNAM Hospital Price Template |
| HPV DNA test | 29.00 | Expert consultation |

### 3.4 Model Parameters

HPVsim includes various categories of parameters, including simulation parameters, demographic parameters, biological parameters, and behavioral parameters. In this appendix, we present the parameters customized for our study, detailed in Appendix Tables 4,5 and 6. Parameters not listed in this appendix were set to their default values.

Appendix Table 4: HPVsim parameters and values used for the simulations 1/2

| **Name** | **Description** | **Value** | **Source** |
|---|---|---|---|
| location | Defines the correct demographic data | Tunisia | Chosen by researcher |
| network | Defines the type of the sexual network | default | Chosen by researcher |
| n_agents | Size of the agent population | 50000 | Check appendix 2 |
| start | Year the simulation starts | 1990 | Chosen by researcher |
| end | Year the simulation ends | 2090 | Chosen by researcher |
| burnin | Duration of the burn-in period of the simulation | 20 | Chosen by researcher |
| rand_seed | Random seed | 1 | Chosen by researcher |
| use_migration | Considers migration's effect | False | Chosen by researcher |
| init_hpv_prev | Initial age distribution of HPV infections | **age_brackets:** [30, 40, 50, 150] **m:** [0.124, 0.07, 0.064, 0.081] **f:** [0.124, 0.07, 0.064, 0.081] | Source [2] |
| init_hpv_dist | Initial HPV genotypes distribution | **hpv31:** 0.019 **hpv16:** 0.011 **hpv59:** 0.0125 **hpv52:** 0.012 **hpv18:** 0.011 **hpv35:** 0.0055 **hpv58:** 0.0065 **hpv56:** 0.0045 **hpv39:** 0.0045 **lr:** 0.9135 | Check appendix 2.2 |

For the probabilities of transition from infection to CIN2+, we used the default values in HPVsim. HPVsim explicitly accounts for genotype-specific transition probabilities for HPV 16 and 18 while grouping other high-risk and low-risk genotypes separately [11].

Regarding the sensitivity and accuracy of the screening tests used in this study (Pap smear cytology and HPV test), we relied on the values from [9, 11]

### 3.5 No-interventions Scenario Simulation

We simulated the no-intervention scenario that consists of having no medical intervention at all, not even the current screening. Then, we assessed the reduction of HPV cases and cancer cases of the Scenarios (1-4) when compared to the no-intervention scenario. This is in order to get insights on the overall burden of the HPV and the gross efficiency of the scenarios tested in this study. The simulation projected a reduction in both HPV infections and cervical cancer cases by 64% and 74%, respectively, by the year 2090 for Scenario 1 which maintains the current screening strategy in Tunisia while introducing the HPV routine vaccination. As for Scenario 2, which introduced HPV DNA testing at a frequency of one test for women aged 35–40 plus routine vaccination, showed reductions in HPV infections and cervical cancer cases by 59-69% and 75-76%, respectively (see Appendix Figures 6a and 6b in blue). Scenario 3, which introduced HPV DNA testing at a frequency of two tests for women aged 35–45 plus routine vaccination, showed reductions in HPV infections and cervical cancer cases by 60-67% and 73-76%, respectively (see Appendix Figures 6a and 6b in yellow). Finally, Scenario 4, which introduced HPV DNA testing at a frequency of every 5 years for women aged 35–60 plus routine vaccination, showed reductions in HPV infections and cervical cancer cases by 61-65% and 74-80%, respectively (see Appendix Figures 6a and 6b in green). We notice that the obtained results when comparing the Scenarios (1-4) to the no-interventions scenario are almost identical to the results obtained when comparing to the Baseline scenario which keeps the current screening strategy, slight differences might be attributed to the stochastic nature of the model and the error introduced by smoothening the obtained curves.

### 3.6 Results Statistics

We present in Appendix Tables 7-10 the resulting statistics from simulating the considered scenarios (1-4) in this study.

## References


[1] Marc Arbyn, Massimo Tommasino, Christophe Depuydt, and Joakim Dillner. Are 20 human papillomavirus types causing cervical cancer? *Journal of Pathology*, 234(4):431–435, 2014.

[2] Monia Ardhaoui, Hejer Letaief, Emna Ennaifer, Souha Bougatef, Thelja Lassili, Rahima Bel Haj Rhouma, Emna Fehri, Kaouther Ouerhani, Ikram Guizani, Myriam Mchela, Karim Chahed, Mohamed Kouni Chahed, Mohamed Samir Boubaker, and Nissaf Bouafif Ben Alaya. The Prevalence,

Genotype Distribution and Risk Factors of Human Papillomavirus in Tunisia: A National-Based Study. *Viruses*, 14(10):1–15, 2022.

[3] Laia Bruni, Ginevra Albero, Beatriz Serrano, Maria Mena, Joaquim J Collado, David Gómez, Josep Muñoz, F Xavier Bosch, and Silvia de Sanjosé. Human Papillomavirus and Related Diseases in Tunisia. Summary Report 10 March 2023. Technical report, ICO/IARC Information Centre on HPV and Cancer (HPV Information Centre), 2023.

[4] Hafedh Chekir and Hedia Belhadj. Enquête sur la recomposition des valeurs en rapport avec la sexualité, la santé sexuelle et reproductive et la relation de genre. Technical report, Groupe Tawhida Ben Cheikh, 2023.

[5] Jacques Ferlay, Morten Ervik, Florencia Lam, Mathieu Laversanne, Mathilde Colombet, Laura Mery, Marion Piñeros, Ariana Znaor, Isabelle Soerjomataram, and Freddie Bray. Global Cancer Observatory: Cancer Today, 2022.

[6] Mariem Garci. Cancer du col de l'utérus: étude épidémiologique multicentrique. Master's thesis, School of Medecine, 2020.

[7] France Guérin-Pace and Hassène Kassar. Le célibat et l'entrée en vie maritale : des transformations dans un cadre normé. In France Guérin-Pace and Hassène Kassar, editors, *Tunisie, l'après 2011*. Ined Éditions, Paris, 2022.

[8] H. Khiari, K. Makni, K. Meddeb, O. Jaidane, and M. Hsairi. Cost-effectiveness of human papillomavirus (hpv) vaccination in tunisia: a modelling study. *BMJ Open*, 2024.

[9] George Koliopoulos, Victoria N. Nyaga, Nancy Santesso, Andrew Bryant, Pierre P.L. Martin-Hirsch, Reem A. Mustafa, Holger Schünemann, Evangelos Paraskevaidis, and Marc Arbyn. Cochrane database of systematic reviews. *Cytology versus HPV testing for cervical cancer screening in the general population*, 2017(8), 2017.

[10] Oumaima Laraj, Beya Benzina, Ahlem Gzara, Amira Kebir, Kaja Abbas, and Slimane Ben Miled. Human papillomavirus vaccination at the national level in Tunisia: a cost-effectiveness analysis using a comparative modeling study. *medRxiv*, 2024.

[11] Robyn M Stuart, Jamie A Cohen, Cliff C Kerr, Prashant Mathur, National Disease Modelling Consortium of India, Romesh G Abeysuriya, Marita Zimmermann, Darcy W Rao, Mariah C Boudreau, Serin Lee, Luojun Yang, and Daniel J Klein. HPVsim: An agent-based model of HPV transmission and cervical disease. *PLOS Computational Biology*, 20:1–17, 2024.

[12] Rachel L Winer, James P Hughes, Qinghua Feng, Sandra O'Reilly, Nancy B Kiviat, King K Holmes, and Laura A Koutsky. Condom Use and the Risk of Genital Human Papillomavirus Infection in Young Women. *New England Journal of Medicine*, 354(25):2645–2654, 2006.

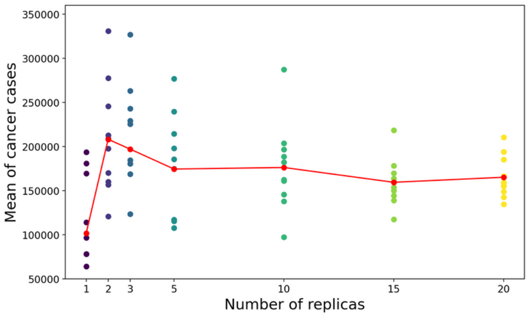


(a) 10000 agents

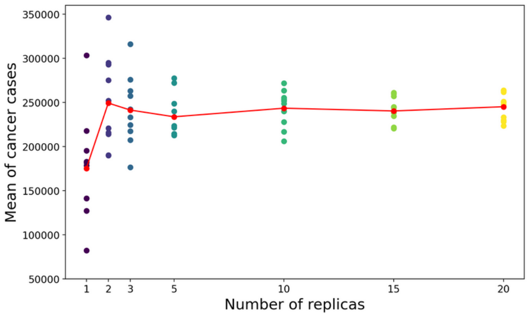


(b) 50000 agents

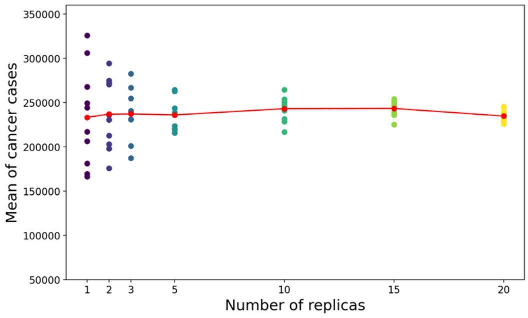


(c) 100000 agents

Appendix Figure 1: The figure presents scatter plots representing the mean cancer cases for each number of replicates across the different agent population sizes. The red curve traces the centroid of each scatter plot (i.e., mean of means).

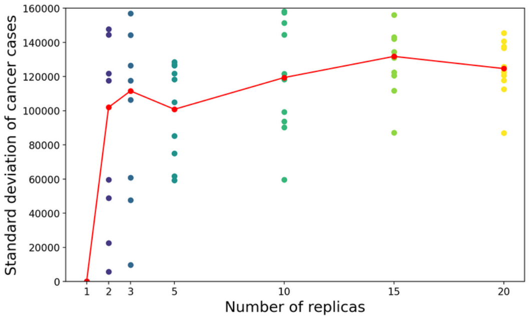


(a) 10000 agents

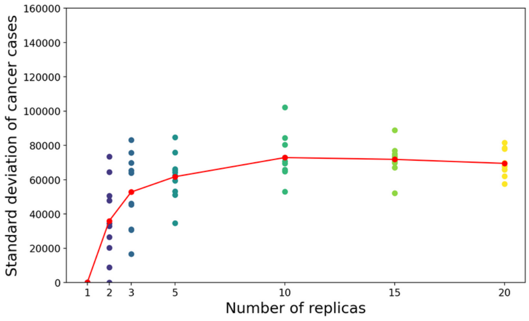


(b) 50000 agents

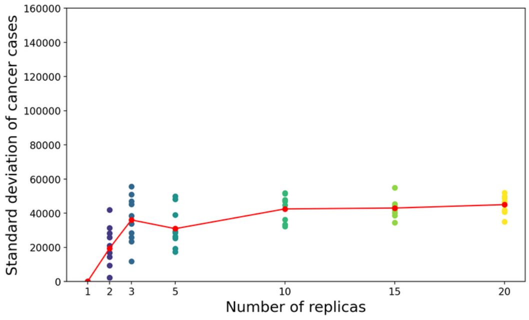


(c) 100000 agents

Appendix Figure 2: The figure presents scatter plots representing the standard deviation of cancer cases of each number of replicates across the different agent population sizes. The red curve traces the centroid of each scatter plot (i.e., mean of standard deviations).

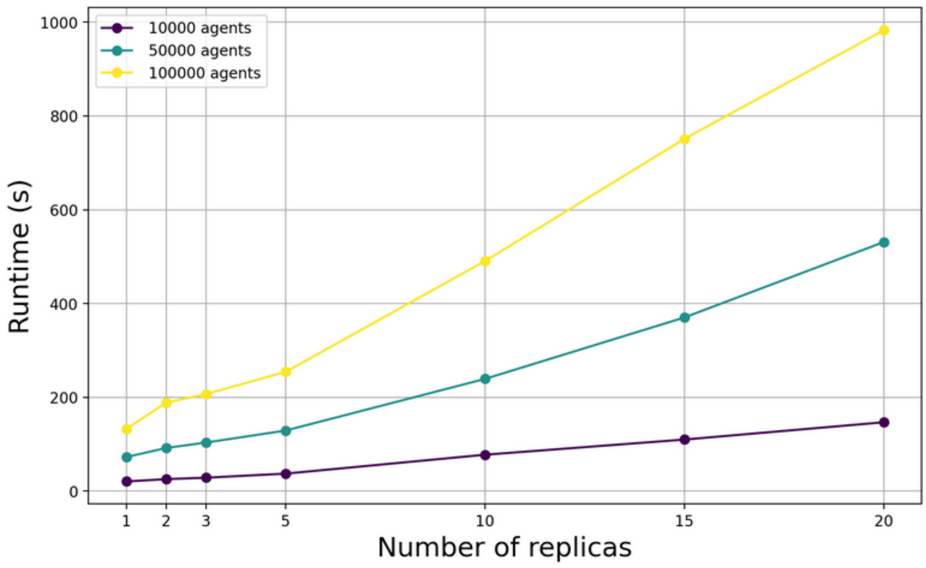


Appendix Figure 3: Running time of simulations for different agents populations sizes as a function of the number of replicates to perform

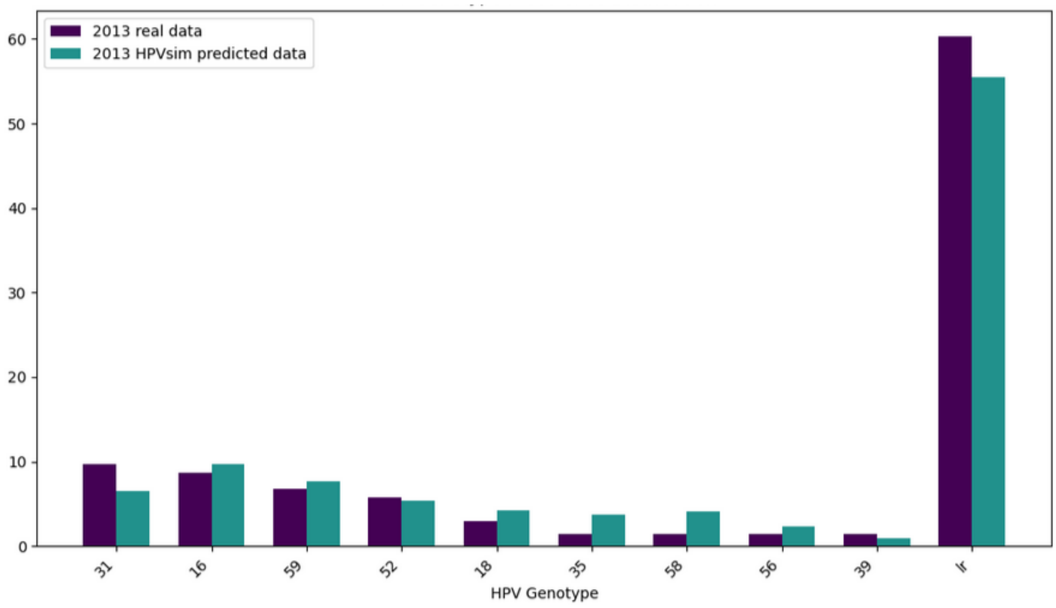


Appendix Figure 4: Barplot of genotype prevalence in the population in 2013, in violet the distribution according to the real data and in green the distribution resulted from the simulation

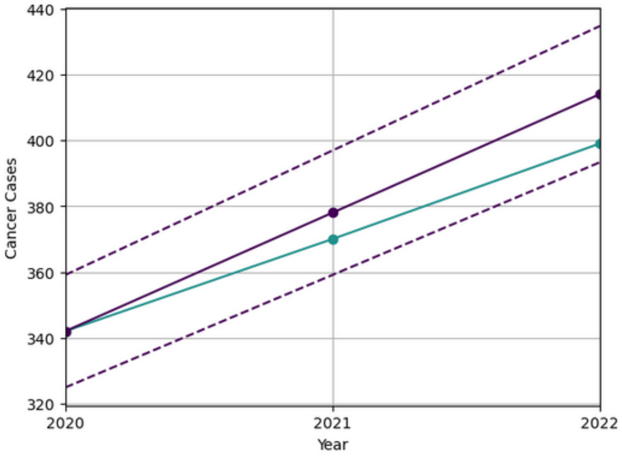


Appendix Figure 5: The figure represent the yearly cancer cases from 2020 to 2022, in violet the real data, in green the predicted yearly cancer cases by HPVsim and the dotted lines represent the 10% error margin

Appendix Table 5: HPVsim parameters and values used for the simulations 2/2

| Name | Description | Value | Source |
|---|---|---|---|
| Debut | Age of sexual debut | **f:** ('normal', par1=23, par2=3)<br>**m:** ('normal', par1=22, par2=4) | Check appendix 2 |
| beta | Transmission coefficient | 0.12 | Calibration value, check appendix 2.2 |
| transm2f | Male-to-female transmission multiplier | 3 | Calibration value, check appendix 2.2 |
| transf2m | Female-to-male transmission multiplier | 1 | Calibration value, check appendix 2.2 |
| eff_condoms | Condom efficiency | 0.7 | Source [12] |
| condoms | Condom usage rate for different types of relationships | **m:** 0.01<br>**c:** 0.18 | Source [4] |
| m_cross_layer | Male cross-layer mixing parameter | 0.25 | Calibration value, check appendix2.2 |
| f_cross_layer | Female cross-layer mixing parameter | 0.1 | Calibration value, check appendix 2.2 |
| hpv_control_prob | Probability of HPV control | 0 | Chosen by researcher |
| dur_pship | Duration of relationships | **m**: ('lognormal', par1=20, par2=26)<br>**c**: ('lognormal', par1=1, par2=2) | Chosen by researcher |
| layer_probs | Share of females and males of each age who are actively seeking relationship if under-partnered | See Excel files: layer_probs_m.xlsx and layer_probs_c.xlsx | Source [7] |
| mixing | Age mixing matrices for marital and casual relationships | See Excel files: mixing_m.xlsx and mixing_c.xlsx | National Institute of Statistics |

Appendix Table 6: Sensitivity and specificity of screening products from [9, 11] appendix

| | VIA | LBC | Pap test | HPV |
|---|---|---|---|---|
| Sensitivity for HSILs | 0.75 | 0.83 | 0.70 | 0.98 |
| Specificity | 0.85 | 0.90 | 0.90 | 0.85 |

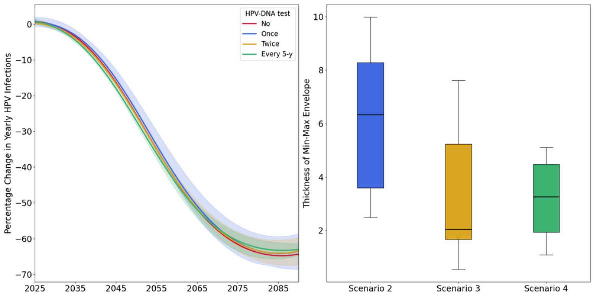


(a) yearly HPV infection cases

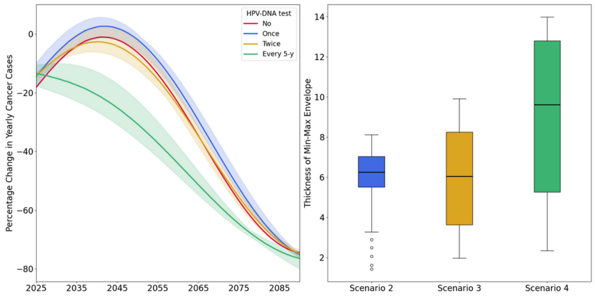


(b) Yearly cancer cases

Appendix Figure 6: **The annual reduction in HPV infections and cancer cases for scenarios (1-4), compared to the no-interventions scenario. For scenarios (2-4), the curves represent the average reductions across various coverage rates tested, with the shaded areas indicating the range of minimum and maximum values (min-max envelope) resulting from different coverage rates. The boxplots display the thickness of the min-max envelope, reflecting the variability due to the different coverage rates tested for each strategy.** Scenario 1 (red): maintaining the current screening strategy coupled with vaccination; Scenario 2 (blue): introducing the HPV DNA test at a frequency of one screening between ages 35 and 40, coupled with vaccination; Scenario 3 (yellow): introducing the HPV DNA test at a frequency of two screenings spaced 5 years apart between ages 35 and 45, coupled with vaccination; Scenario 4 (green): introducing the HPV DNA test at a frequency of every 5 years for women aged 35-60, coupled with vaccination.

Appendix Table 7: Cancers per infection and positive screenings per screening test over time for Scenario 1

| Coverage Rate of HPV screening | Metric (average) | 2025-2034 | 2035-2044 | 2045-2054 | 2055-2064 | 2065-2074 | 2075-2084 | 2084-2090 |
|---|---|---|---|---|---|---|---|---|
| 0 | Positive screenings per screening test | 0.01185 | 0.0189 | 0.01994 | 0.01803 | 0.01068 | 0.00932 | 0.00956 |
| 0 | Cancers per infection | 0.00178 | 0.00303 | 0.0044 | 0.00537 | 0.00527 | 0.00422 | 0.00359 |

Appendix Table 8: Cancers per infection and positive screenings per screening test over time for Scenario 2

| Coverage Rate of HPV screening | Metric (average) | 2025-2034 | 2035-2044 | 2045-2054 | 2055-2064 | 2065-2074 | 2075-2084 | 2084-2090 |
|---|---|---|---|---|---|---|---|---|
| 15% | Positive screenings per screening test | 0.01482 | 0.01505 | 0.01113 | 0.00178 | 0.00035 | 0.00012 | 0.00005 |
| 15% | Cancers per infection | 0.00182 | 0.00297 | 0.00425 | 0.00523 | 0.00532 | 0.00437 | 0.0034 |
| 25% | Positive screenings per screening test | 0.01516 | 0.01701 | 0.01127 | 0.0018 | 0.00053 | 0.00007 | 0.00004 |
| 25% | Cancers per infection | 0.00183 | 0.00302 | 0.00433 | 0.00531 | 0.0053 | 0.00427 | 0.00343 |
| 33% | Positive screenings per screening test | 0.01522 | 0.01773 | 0.01107 | 0.00163 | 0.00056 | 0.00012 | 0.00002 |
| 33% | Cancers per infection | 0.00179 | 0.00298 | 0.00429 | 0.00532 | 0.00541 | 0.00443 | 0.00359 |
| 50% | Positive screenings per screening test | 0.01593 | 0.01706 | 0.00961 | 0.00165 | 0.00045 | 0.00005 | 0.00005 |
| 50% | Cancers per infection | 0.00183 | 0.00304 | 0.00437 | 0.00536 | 0.00537 | 0.00436 | 0.00358 |
| 70% | Positive screenings per screening test | 0.01589 | 0.0165 | 0.00919 | 0.00179 | 0.00025 | 0.00005 | 0.0 |
| 70% | Cancers per infection | 0.00176 | 0.00295 | 0.00427 | 0.00529 | 0.00539 | 0.00451 | 0.00377 |

Appendix Table 9: Cancers per infection and positive screenings per screening test over time for Scenario 3

| Coverage Rate of HPV screening | Metric (average) | 2025-2034 | 2035-2044 | 2045-2054 | 2055-2064 | 2065-2074 | 2075-2084 | 2084-2090 |
|---|---|---|---|---|---|---|---|---|
| 15% | Positive screenings per screening test | 0.01732 | 0.01923 | 0.0169 | 0.00367 | 0.00073 | 0.00012 | 0.0 |
| 15% | Cancers per infection | 0.00179 | 0.003 | 0.00432 | 0.0053 | 0.00532 | 0.00441 | 0.00379 |
| 25% | Positive screenings per screening test | 0.01719 | 0.02083 | 0.01696 | 0.00332 | 0.00085 | 0.00022 | 0.00004 |
| 25% | Cancers per infection | 0.00181 | 0.00295 | 0.0042 | 0.00511 | 0.00506 | 0.00413 | 0.00346 |
| 33% | Positive screenings per screening test | 0.01858 | 0.02042 | 0.01578 | 0.00353 | 0.00076 | 0.0009 | 0.00002 |
| 33% | Cancers per infection | 0.0018 | 0.00285 | 0.00401 | 0.00496 | 0.00515 | 0.00442 | 0.00367 |
| 50% | Positive screenings per screening test | 0.01906 | 0.02165 | 0.01792 | 0.00346 | 0.0007 | 0.00012 | 0.00005 |
| 50% | Cancers per infection | 0.00175 | 0.00286 | 0.00407 | 0.00492 | 0.00483 | 0.00395 | 0.00345 |
| 70% | Positive screenings per screening test | 0.01964 | 0.02088 | 0.01504 | 0.00249 | 0.00046 | 0.00013 | 0.0 |
| 70% | Cancers per infection | 0.00175 | 0.00279 | 0.00395 | 0.00487 | 0.00497 | 0.00414 | 0.00334 |

Appendix Table 10: Cancers per infection and positive screenings per screening test over time for Scenario 4

| Coverage Rate of HPV screening | Metric (average) | 2025-2034 | 2035-2044 | 2045-2054 | 2055-2064 | 2065-2074 | 2075-2084 | 2084-2090 |
|---|---|---|---|---|---|---|---|---|
| 15% | Positive screenings per screening test | 0.01873 | 0.02287 | 0.02306 | 0.01235 | 0.00283 | 0.00046 | 0.0009 |
| 15% | Cancers per infection | 0.00173 | 0.0026 | 0.00354 | 0.00428 | 0.00433 | 0.00369 | 0.00321 |
| 25% | Positive screenings per screening test | 0.01936 | 0.02389 | 0.02418 | 0.01213 | 0.0032 | 0.00046 | 0.00009 |
| 25% | Cancers per infection | 0.00166 | 0.00247 | 0.0033 | 0.00389 | 0.00391 | 0.00349 | 0.00336 |
| 33% | Positive screenings per screening test | 0.01906 | 0.02311 | 0.02177 | 0.01071 | 0.00276 | 0.00028 | 0.00008 |
| 33% | Cancers per infection | 0.00167 | 0.00237 | 0.0031 | 0.00365 | 0.00371 | 0.00343 | 0.00336 |
| 50% | Positive screenings per screening test | 0.01965 | 0.02231 | 0.02 | 0.00951 | 0.00229 | 0.00033 | 0.00013 |
| 50% | Cancers per infection | 0.00162 | 0.0023 | 0.00301 | 0.00352 | 0.00354 | 0.0032 | 0.00308 |
| 70% | Positive screenings per screening test | 0.01956 | 0.02357 | 0.02197 | 0.01089 | 0.00272 | 0.00032 | 0.0001 |
| 70% | Cancers per infection | 0.00161 | 0.00226 | 0.00302 | 0.00372 | 0.00395 | 0.00352 | 0.00298 |
